# COVID-19 Infection Risk Among Vulnerable Healthcare Workers: The Protective Role of Pre-Pandemic Recognition

**DOI:** 10.3390/clinpract16030048

**Published:** 2026-02-26

**Authors:** Maria Ladisa, Juan Luís Cabanilla-Moruno, Lara Estefanía Jiménez-Ortega, Manuel Delgado-Calderón, Emilio García-Cabrera, Julia Romero-Barranca, Ángel Vilches-Arenas

**Affiliations:** 1 Department of Preventive Medicine, Hospital Universitario Virgen Macarena, 41009 Seville, Spain; maria.ladisa.sspa@juntadeandalucia.es (M.L.); larae.jimenez@juntadeandalucia.es (L.E.J.-O.); manuel.delgado.calderon.sspa@juntadeandalucia.es (M.D.-C.); ava@us.es (Á.V.-A.); 2 Department of Preventive Medicine and Public Health, University of Seville, 41009 Seville, Spain; jlcabanillas@us.es (J.L.C.-M.); julia.romero.barranca.sspa@juntadeandalucia.es (J.R.-B.); 3 Occupational Risk Prevention Unit, Hospital Universitario Virgen Macarena, 41009 Seville, Spain

**Keywords:** COVID-19, SARS-CoV-2, healthcare workers, occupational health, vulnerable workers, pandemic, risk prevention

## Abstract

**Background:** During the first wave of the COVID-19 pandemic, the importance of the recognition of vulnerable workers was well-established, but the specific impact of the timing of their recognition remains less understood. **Objective**: This study evaluates the impact of early recognition of vulnerable healthcare workers (VHCWs) and identifies factors associated with SARS-CoV-2 infection. **Methods**: We performed a retrospective cohort study at the Virgen Macarena University Hospital (HUVM) in Seville and included employees classified as VHCWs between January 2020 and December 2021. All data, including demographic, occupational, and clinical data, were collected from occupational health records and the Andalusian digital health system. The incidence of COVID-19 was analyzed using descriptive, bivariate statistics, and Cox regression. **Results**: A total of 471 VHCWs were included. Most of the VHCWs were women (79.8%) with a median age of 50 years. The most common vulnerability criteria were pregnancy (32.9%) and age > 60 (28.7%). During the study period, 58 VHCWs (12.3%) were diagnosed with COVID-19, compared to 18.35% of the general workforce. Recognition of VHCW status after the pandemic was declared was strongly associated with higher infection risk (HR = 48.84; 95% CI: 26.21–90.99; *p* < 0.001). **Conclusions**: The timing of vulnerability recognition emerged as the most critical protective factor in this cohort. Healthcare workers whose vulnerability was not proactively identified before the pandemic onset faced a substantially higher risk of infection (HR = 44.68; 95% CI: 26.21–90.99; *p* < 0.001) compared to those recognized early. These findings underscore that pre-pandemic identification facilitated the immediate implementation of task adaptations and workplace restrictions, effectively mitigating high-risk exposure during the most critical early stages of the crisis.

## 1. Introduction

In late 2019, the appearance of a new coronavirus, SARS-CoV-2 [1,2], marked the beginning of a global crisis. The epidemic reached its peak in China between January and February 2020 [3], while in Europe, the first major outbreak was recorded in Italy in late February. The rapid spread of the resulting disease, COVID-19, led the World Health Organization (WHO) to declare a pandemic on 11 March 2020 [4]. In response, nations around the world, including Spain [5], implemented measures that were unprecedented in recent healthcare history, such as national lockdowns to curb transmission.

Healthcare workers (HCWs) were the first line of the pandemic response. They faced a disproportionate risk of exposure to infection with a wide range of clinical manifestations, from asymptomatic cases to multi-organ failure [6]. Certain workers were identified as being particularly vulnerable to severe outcomes due to factors such as advanced age, pre-existing medical conditions, immunosuppression, or pregnancy [7,8,9,10]. The protection of these Vulnerable Health Care Workers (VHCWs) became a critical priority for healthcare systems. The rationale for prioritizing these workers was based on early reports from Wuhan and Italy indicating that comorbidities such as hypertension and diabetes were strongly associated with ICU admission and mortality. Consequently, the precautionary principle led to the immediate withdrawal or relocation of these staff members, even before robust epidemiological evidence on occupational transmission was fully available. In response to this situation, occupational health services played a pivotal role in the management of infection within the workplace by regulating preventive measures and protocols for workers.

In Spain, the Ministry of Health established a national protocol on 5 March 2020 (continuously updated throughout the pandemic) for the prevention [10,11], which guided the identification and management of VHCWs. Vulnerability was clinically defined as workers with diabetes, cardiovascular disease (hypertension included), chronic lung disease, immunodeficiency, cancer under active treatment, pregnancy, and age over 60 years. These measures included risk assessments, workplace adaptations, and, where necessary, temporary removal from high-exposure duties. Although the importance of vulnerable workers is well-established, the impact of the timing of the formal recognition of VHCW status remains less understood. It is unclear whether proactive, pre-pandemic identification of at-risk individuals conferred greater protection than identification that occurred after the pandemic was officially declared.

Therefore, this study aims to conduct a retrospective analysis to evaluate the protective effect of early recognition of VHCWs at the HUVM. We seek to identify key risk factors associated with SARS-CoV-2 infection within this vulnerable cohort and determine if the pre-pandemic classification was a significant protective factor.

## 2. Materials and Methods

### 2.1. Study Design and Cohort Definition

An analytical retrospective cohort study was conducted at the Hospital Universitario Virgen Macarena (HUVM) in Seville, Spain. The study period spanned from 1 January 2020 to 31 December 2021.

Inclusion criteria for the cohort were: (1) being an active healthcare worker at the HUVM during the study timeframe; (2) having a minimum employment duration of three months to ensure consistent occupational exposure; and (3) being formally recognized as a vulnerable healthcare worker (VHCW) by the Occupational Risk Prevention Unit. Following national clinical guidelines [7,8,9,10], vulnerability was defined by the presence of at least one of the following conditions: arterial hypertension, cardiovascular disease, chronic pulmonary disease, diabetes, chronic kidney disease, chronic liver disease, clinical immunosuppression, active neoplasia, obesity (BMI > 40 kg/m^2^), or being over 60 years of age.

Exclusion criteria were applied to maintain cohort homogeneity and minimize confounding: (1) workers who had retired prior to the study start date; and (2) workers whose vulnerability was solely based on pregnancy or breastfeeding. These groups were excluded due to the specific and evolving administrative protections and legal frameworks that applied to them during the pandemic, which differed from clinical comorbidities, as well as the limited early evidence regarding infection susceptibility in these specific groups [12]. The final cohort was assembled by cross-referencing the Occupational Risk Prevention Unit clinical registries with the hospital’s human resources database to verify employment status and the exact date of vulnerability recognition. This study was carried out following the STROBE guideline [13].

### 2.2. Data Collection

Data collected were extracted retrospectively from the Occupational Health and Safety Department’s electronic records, stored in the Occupational Health software of the Andalusian Health Service WinMEDTRA^®^ (version 11) [14]. Clinical variables regarding COVID-19 infection (symptoms, dates of diagnosis) were verified through the hospital’s Preventive Medicine epidemiological surveillance system, and the unified digital health record system of the Andalusian Public Health System, Diraya [15]. The data extraction process commenced after receiving formal approval from the Research Ethics Committee of the Virgen Macarena and Virgen del Rocio University Hospitals on 19 July 2021. Informed consent was obtained from all participating workers for access to their health data and participation in the study. The data collected included sociodemographic and occupational characteristics: sex, age, level of education, living arrangements, professional category, job position, length of service, and working hours. In addition, data were recorded on the recognition of VHCW status and work-related variables linked to COVID-19. These data include employment in high-risk COVID-19 areas, use of personal protective equipment (PPE), reasons and pathways for VHCW recognition, outcomes of the occupational health assessment, work adaptations made, COVID-19 vaccination status, and associated side effects.

For individuals who tested positive for COVID-19, data collected were the type of active infection diagnostic test (PDIA), nature of exposure/contact, symptoms, clinical classification, hospitalization requirements, clinical progression, and duration of illness.

### 2.3. Exposure and Outcome Definitions

The key exposure was the timing of vulnerability recognition. “Recognized before pandemic onset” was defined as workers already identified as VHCWs in their occupational health records prior to 14 March 2020 (the start of the national state of alarm in Spain). “Recognized after” refers to those whose clinical vulnerability was identified during the pandemic through a specific request or screening. The primary outcome was the first confirmed SARS-CoV-2 infection, defined by a positive PDIA (RT-PCR or rapid antigen test) recorded in the hospital’s epidemiological surveillance system. Only the first event per worker was considered to calculate the incidence.

### 2.4. Data Analysis

A descriptive analysis was conducted. Quantitative variables were summarized using means and standard deviations or medians and interquartile ranges (IQR: p25–p75), depending on the distribution of the data. Qualitative variables were summarized using frequency tables and percentages. All point estimates were accompanied by 95% confidence intervals, which is a more appropriate method of determining statistical power [16].

A bivariate analysis was performed to evaluate the characteristics within the VHCW cohort that were associated with acquiring a COVID-19 infection. Pearson’s Chi-square test, Chi-square with continuity correction, or Fisher’s exact test were used as appropriate for qualitative variables. For quantitative variables, after verifying the assumptions of homoscedasticity, independence of observations, and normality of distributions, Student’s *t*-test was applied; if the normality assumption was not met, the Mann–Whitney U test was used.

Univariate survival analysis was performed using Kaplan–Meier curves to estimate the time to the first positive PDIA (dependent variable) based on the study’s primary independent variable: the timing of vulnerability recognition. Cumulative probabilities of COVID-19 infection were calculated throughout the follow-up period, along with median survival times and interquartile ranges (IQR; P25–P75) with their corresponding 95% confidence intervals (CI). Survival functions were stratified and compared between groups (recognized before vs. after pandemic onset) and by clinical comorbidities. The Log-Rank test was used to evaluate the equality of survival distributions between the different cohorts. Cox proportional hazards regression models were employed to identify predictors of time to the first positive PDIA, incorporating both categorical and continuous covariates. Variable selection for the multivariable model followed a two-stage process: first, a preliminary univariable analysis identified variables associated with the outcome at a significance level of *p* < 0.15; subsequently, a multivariable Cox regression was performed to determine the independent effect of each factor while controlling for potential confounders. The proportional hazards assumption was verified for all models. Results are reported as Hazard Ratios (HR) with 95% CIs.

All analyses were conducted assuming a type I error of 5% and a type II error of 20%. The statistical software used was IBM SPSS Statistics, version 29.

## 3. Results

During the study period, the HUVM workforce was composed of 6019 professionals; of these, 4159 were healthcare workers (15.2% doctors, 5.2% resident interns, 27.8% nurses, and 21% nursing assistants). The remaining 1860 members of the workforce were composed of orderlies, general services personnel, including kitchen staff, maintenance workers, cleaners, laundry staff, and specialist technicians (Table 1).

A total of 471 workers were recognized as VHCWs. Of the sample, 79.8% were women and 20.2% were men. The median age was 50 years (IQR: 36–60), ranging from 23 to 69 years, with a median job tenure of 178 months (IQR: 108–197) and a median tenure in the current position of 150 months (IQR: 59–179). A total of 33 workers (7%) recognized as VHCWs also had a disability.

Hypertension was observed in 34.2% of the sample, diabetes was present in 17% of the participants, and dyslipidemia in 13.4%. A total of 155 women workers (32.9%) were classified as especially vulnerable due to pregnancy, and 135 workers (28.7%) due to being over 60 years of age (Table 2).

During the study period, 1105 COVID-19 infections were observed among HUVM workers, of whom 58 were VHCWs. The cumulative incidence in health workers was 18.35 per 100 workers and 12.31 per 100 workers in the VHCW group. All COVID-19 infections required temporary invalidity (TI), and in VHCW groups, 383 (81.3%) TI, 243 (63.4%) were directly related to the COVID-19 process.

The analysis of the risk factors for COVID-19 in VHCWs with infection is shown in Table 3. We did not find any risk factors associated with COVID-19 infections; note that 13 workers (22.4%) with COPD were affected, versus 51 (12.3%) workers without COVID-19 infection. Additionally, a higher infection rate was observed among workers younger than 60 years; 48 workers (82.2%) versus 288 (69.9%).

A total of 40 VHCWs (8.5%) were recognized for their special condition after declaring the pandemic state. Of these, 20 (50%) had COVID-19 infection (*p* < 0.001 in the multivariate analysis, two variables remained significant). We analyzed specific comorbidities (diabetes, hypertension, obesity, etc.) individually. None of the specific pathologies showed a statistically significant association with a higher risk of infection in the multivariate analysis. Recognition of VHCW status after the pandemic was declared was the strongest risk factor for infection (HR = 48.84; 95% CI: 26.21–90.99; *p* < 0.001). Conversely, age over 60 was identified as a protective factor (HR = 0.51; 95% CI: 0.27–0.96; *p* = 0.036), as shown in Table 4.

## 4. Discussion

The main result of this study highlights the extraordinary importance of occupational medicine services during the COVID-19 pandemic. The late formal recognition of a healthcare worker’s vulnerable status increased their risk of infection by nearly 49 times. This risk increase reflects the direct consequence of unprotected occupational exposure during the pandemic’s initial phase. During this period, characterized by uncertainty and widespread PPE shortages [17,18,19,20,21], these individuals continued their duties in high-risk environments, compared to their peers who had already been reassigned to low-exposure roles, underscoring the role of proactive and timely intervention [21].

This proactive management would have effectively neutralized their occupational risk, demonstrating that workplace adaptations can override an individual’s vulnerability. An apparently paradoxical result of our study is that while traditional risk factors like comorbidities [19] showed no significant association with infection, age over 60 emerged as a protective factor. Far from contradicting established clinical findings, this finding likely illustrates the effectiveness of the individualized preventive measures that were implemented. Workers in the highest-risk categories, such as those over 60, were prioritized for protective actions, including immediate reassignment to low-risk areas or telework [10,11].

Notably, the cumulative incidence of COVID-19 among the vulnerable healthcare worker (VHCW) cohort was lower than that of the general hospital staff (12.31% vs. 18.35%). This result—where clinically higher-risk individuals experienced lower infection rates than the overall workforce—demonstrates the high efficacy of targeted occupational preventive protocols. The data suggest that rigorous task adaptation and the reassignment of VHCWs to low-exposure areas successfully neutralized their inherent clinical susceptibility, rendering them less occupationally vulnerable than their healthy counterparts in high-exposure roles. In our hospital, the rate was lower than that of the general hospital staff and comparable to rates reported in similar Spanish healthcare canters during the same period [20,21,22,23]. However, the relatively low number of total infections in our cohort may have limited the statistical power needed to detect associations for less prevalent conditions. This, along with a potential selection bias where early interventions attenuated observable risks, could also contribute to the lack of association with traditional risk factors or specific comorbidities [19,24].

Our findings reaffirm the role played during the pandemic by occupational health services. Their function extended far beyond issuing fitness-for-work reports; they were instrumental in managing job adaptations, coordinating with prevention specialists, strengthening vaccination campaigns, and providing essential psychological support [19,25]. This was made despite significant challenges, including limited resources and the immense psychological toll of the early pandemic waves [26,27]. The ability of these services to successfully protect the most vulnerable workers underscores their strategic value in maintaining the resilience of the healthcare workforce [19].

This study has several limitations that should be acknowledged. First, its retrospective design introduces potential biases related to data collection and the reliance on historical records. Second, a critical limitation was the testing strategy employed during the early phase of the pandemic. Due to the global scarcity of diagnostic resources, PCR testing was primarily restricted to symptomatic personnel. Consequently, asymptomatic infections may have gone undetected, leading to a potential underestimation of the true infection rate across the entire workforce. However, this symptom-based criterion was applied universally, minimizing the risk of differential surveillance bias between vulnerable and non-vulnerable groups.

Third, regarding the multivariate analysis, the high magnitude of the risk estimate (HR = 48.84) for late recognition should be interpreted with caution. This elevated figure, along with the wide confidence intervals, is likely influenced by the relatively low number of infection events observed in the early-recognized VHCW cohort. Finally, the scarcity of literature specifically regarding pre-classified vulnerable workers limits the possibilities for external validation. Further prospective studies with larger sample sizes and routine screening protocols are needed to confirm this association and more precisely quantify the protective value of early occupational health interventions.

## 5. Conclusions

In conclusion, there is a clear need to integrate occupational risk prevention comprehensively within healthcare organizations. The current model should evolve from a reactive to a proactive one, where initial and periodic medical examinations are used to systematically maintain a registry of vulnerable workers. This would allow for the rapid identification and protection of at-risk employees in future pandemics or health crises. Developing structural strategic plans to address these systemic shortcomings is therefore essential for safeguarding our healthcare professionals. The pandemic experience validates the critical necessity of institutionalizing pre-employment health screenings as a cornerstone of ‘preventive intelligence. Beyond fulfilling legal mandates, comprehensive clinical baseline assessments enable dynamic risk stratification. This proactive approach ensures that, during a public health emergency, vulnerability status can be immediately activated within hospital management systems, allowing for instantaneous protective measures and bypassing the administrative delays that characterized the early response to SARS-CoV-2.

## Figures and Tables

**Table 1:** Labor characteristics of hospital workers.

Professional Category	*N* (%)	95% CI
Specialist Doctor	918 (15.2)	(14.3; 16.1)
Resident Internal Specialist	313 (5.2)	(4.6; 5.8)
Nurse	1681 (27.8)	(26.7; 29.0)
Nursing Assistant	1247 (21.0)	(20.0; 22.0)
Orderly	492 (8.1)	(7.5; 8.9)
General Services Staff	818 (13.5)	(12.7; 14.4)
Specialized Technicians	550 (9.1)	(8.4; 9.9)
Job position		
Medical Area	2006 (33.2)	(32.0; 34.4)
Medical-Surgical Area	476 (7.9)	(7.3; 8.5)
Surgical Area	686 (11.3)	(10.5; 12.2)
Medical Services Area	578 (9.6)	(8.8; 10.4)
Anesthesia/ICU	344 (5.7)	(5.1; 6.3)
Emergency	405 (6.7)	(6.1; 7.3)
On Call/Reserve	753 (12.5)	(11.6; 13.4)
General Services	791 (13.1)	(12.2; 14.0)

**Table 2:** Sociodemographic and labor characteristics of VHCWs.

Variable	Category	*N*	Value	95% CI
Age Median (p25, 75)	-	471	50.0 (36.0; 60.0)	(47.0; 54.0)
Sex (%)	Men	95	20.2	(16.7; 24.0)
	Women	376	79.8	(76.0; 83.3)
Length of service (months) Median (p25, 75)	-	471	178.0 (108.0; 197.0)	(178.0; 180.0)
Professional Category (%)	Specialist Doctor	92	19.5	(16.1; 23.3)
	Resident Internal Specialist	5	1.1	(0.4; 2.3)
	Nurse	142	30.1	(26.1; 34.4)
	Nursing Assistant	130	27.6	(23.7; 31.8)
	Orderly	25	5.3	(3.6; 7.6)
	General Services Staff	54	11.5	(8.8; 14.6)
	Specialized Technicians	23	4.9	(3.2; 7.1)
	Medical Area	149	31.6	(27.5; 36.0)
Job Position (%)	Medical-Surgical Area	48	10.2	(7.6; 13.4)
	Surgical Area	33	7.0	(4.9; 9.9)
	Medical Services Area	34	7.2	(5.0; 10.1)
	Anesthesia/ICU	32	6.8	(4.7; 9.6)
	Emergency	60	12.7	(9.8; 16.1)
	On Call/Reserve	33	7.0	(4.9; 9.9)
	General Services	63	13.4	(10.5; 16.8)
COVID-19 Work Zone	Yes	87	18.5	(15.4; 22.4)
	No	383	81.5	(77.6; 84.6)
Use of PPE (%) ^1^	Gloves	462	98.1	(96.5; 99.1)
	Surgical Mask	467	99.2	(98.0; 99.7)
	FFP2 Mask	446	94.7	(92.4; 96.4)
	FFP3 Mask	237	50.3	(45.8; 54.8)
	Face shield	130	27.6	(23.7; 31.8)
	Glasses	309	65.7	(61.4; 69.9)
	Disposable gowns	261	55.4	(50.9; 59.9)
Condition for VHCWs Recognition (%) ^1^	Cardiovascular Disease	64	13.6	(10.7; 16.9)
	Hypertension	124	26.3	(22.5; 30.4)
	Chronic Pulmonary Disease	64	13.6	(10.7; 16.9)
	Diabetes	66	14.0	(11.1; 17.4)
	Severe Chronic Hepatic D.	20	4.2	(2.7; 6.4)
	Chronic Renal Insufficiency	6	1.3	(0.5; 2.6)
	Immunosuppression	50	10.6	(8.1; 13.6)
	Active Cancer	21	4.5	(2.9; 6.6)
	Morbid Obesity	27	5.7	(3.9; 8.1)
	Pregnancy	155	32.9	(29.4; 37.9)
	Over 60 years old	135	28.7	(24.7; 32.9)
Route for VHCW Recognition	Initial Exam	3	0.6	(0.2; 1.7)
	Periodic Exam	5	1.1	(0.4; 2.3)
	Special Exam	308	65.4	(61.0; 69.6)
	Pregnant Workers	155	32.9	(28.8; 37.2)

^1^ Multiple responses were allowed.

**Table 3:** Risk factors of COVID-19 infection in VHCWs.

		COVID-19 Infection *N* = 58	95% CI	COVID-19 Non-Infection *N* = 325	95% CI	*p*
Age (years) Median (p25, 75)		46.0 (36.0; 57.0)	[41.0; 52.0]	51.0 (36.0; 61.0)	[47.0; 54.0]	0.292
Age over 60	No	48 (82.8)	[71.6; 90.8]	288 (69.7)	[65.2; 74.0]	0.058
	Yes	10 (17.2)	[9.2; 28.4]	125 (30.3)	[26.0; 34.8]	
Sex *n* (%)	Men	12 (20.7)	[11.8; 32.4]	83 (20.1)	[16.4; 24.2]	0.999
	Women	46 (79.3)	[67.6; 88.2]	330 (79.9)	[75.8; 83.6]	
BMI (kg/m^2^) Median (p25, 75)		25.3 (21.9; 28.9)	[24.2; 26.2]	25.6 (22.8; 29.4)	[25.2; 26.2]	0.464
BMI over 30 *n* (%)	No	56 (96.6)	[89.4; 99.3]	388 (93.9)	[91.3; 95.9]	0.619
	Yes	2 (3.4)	[0.7; 10.6]	25 (6.1)	[4.1; 8.7]	
Vaccination *n* (%)	No	3 (5.2)	[1.5; 13.2]	8 (1.9)	[0.9; 3.6]	0.288
	Yes	55 (94.8)	[86.8; 98.5]	405 (98.1)	[96.4; 99.1]	
Cardiovascular disease *n* (%)	No	55 (94.8)	[86.8; 98.5]	352 (85.2)	[81.6; 88.4]	0.073
	Yes	3 (5.2)	[1.5; 13.2]	61 (14.8)	[11.6; 18.4]	
Hypertension *n* (%)	No	44 (75.9)	[63.8; 85.4]	303 (73.4)	[68.9; 77.5]	0.806
	Yes	14 (24.1)	[14.6; 36.2]	110 (26.6)	[22.5; 31.1]	
COPD *n* (%)	No	45 (77.6)	[65.7; 86.8]	362 (87.7)	[84.2; 90.6]	0.059
	Yes	13 (22.4)	[13.2; 34.3]	51 (12.3)	[9.4; 15.8]	
Diabetes Mellitus *n* (%)	No	47 (81.0)	[69.6; 89.5]	358 (86.7)	[83.2; 89.7]	0.338
	Yes	11 (19.0)	[10.5; 30.4]	55 (13.3)	[10.3; 16.8]	
Chronic Hepatic Disease *n* (%)	No	55 (94.8)	[86.8; 98.5]	396 (95.9)	[93.6; 97.5]	0.979
	Yes	3 (5.2)	[1.5; 13.2]	17 (4.1)	[2.5; 6.4]	
CRD *n* (%)	No	57 (98.3)	[92.2; 99.8]	408 (98.8)	[97.4; 99.5]	0.548
	Yes	1 (1.7)	[0.2; 7.8]	5 (1.2)	[0.5; 2.6]	
Immunosuppression *n* (%)	No	52 (89.7)	[79.9; 95.6]	369 (89.3)	[86.1; 92.0]	0.999
	Yes	6 (10.3)	[4.4; 20.1]	44 (10.7)	[8.0; 16.9]	
Cancer *n* (%)	No	58 (100)		392 (94.9)	[92.5; 96.7]	0.156
	Yes	0 (0)		21 (5.1)	[3.3; 7.5]	
Pregnancy *n* (%)	No	42 (72.4)	[60.0; 82.6]	271 (65.6)	[60.9; 70.1]	0.380
	Yes	16 (27.6)	[17.4; 40.0]	142 (34.4)	[29.9; 39.1]	
Week of pregnancy Median (p25, 75)		8.5 (6.5; 12.5)	[7.0; 12.0]	11.0 (7.0; 16.0)	[10.0; 13.0]	0.205
Recognition as VHCWs *n* (%)	B.P.	38 (65.5)	[53.4; 76.5]	451 (95.8)	[93.5; 97.2]	<0.001
	A.P.	20 (34.5)	[24.2; 47.3]	20 (4.2)	[2.9; 6.8]	

COPD: chronic obstructive pulmonary disease; CRD: chronic renal disease; B.P.: before pandemic onset; A.P.: after pandemic onset.

**Table 4:** Risk factors of COVID-19 in VHCWs.

UNIVARIATE ANALYSIS		HR	95% CI	*p*
Sex	Man	1		
	Woman	0.96	0.51; 1.81	0.905
Age	Under 60 years old	1		
	Over 60 years old	0.65	0.35; 1.20	0.171
Disability	No	1		
	Yes	0.99	0.36; 2.76	0.997
Incapacity	No	1		
	Yes	0.05	0.0; 9358.39	0.627
Vaccination	No	1		
	Yes	0.41	0.13; 1.31	0.133
Full vaccination course	No	1		
	Yes	1.17	0.55; 2.47	0.683
Recognition as VHCWs	Declared before the pandemic	1		
	Declared after the pandemic	44.68	24.14; 82.73	<0.001
Professional Category	Non-healthcare	1		
	Healthcare	1.18	0.61; 2.28	0.615
Position	Medical area	1		
	Surgical area	1.09	0.63; 1.88	0.765
	Administration	1.02	0.42; 2.46	0.961
**MULTIVARIATE ANALYSIS**		**HR**	**95% CI**	***p***
Age	Under 60 years old	1		
	Over 60 years old	0.51	0.27; 0.96	0.036
Recognition as VHCWs	Declared before	1		
	Declared after	48.84	26.21; 90.99	<0.001

## Data Availability

The data that support the findings of this study are available from the authors, but restrictions apply to the availability of these data, which were used under license from the Andalusian Health Service (Seville) for the current study, and so are not publicly available. Data are, however, available from the authors upon reasonable request and with permission from the Andalusian Health Service.

## Abbreviations

The following abbreviations are used in this manuscript:

AP	After pandemic
BMI	Body mass index
BP	Before pandemic
COPD	Chronic Obstructive Pulmonary Disease
CRD	Chronic Renal Disease
HCWs	Healthcare workers
HUVM	Virgen Macarena University Hospital
ICU	Intensive Care Unit
IQR	Interquartile range
PDIA	Positive active infection diagnostic
PPE	Personal protective equipment
TI	Temporary invalidity
VHCWs	Vulnerable Health Care Workers
WHO	World Health Organization
